# Veno-Arterial Extracorporeal Membrane Oxygenation in Patients with Fulminant Myocarditis: A Review of Contemporary Literature

**DOI:** 10.3390/medicina58020215

**Published:** 2022-02-01

**Authors:** Shreyas Venkataraman, Abhishek Bhardwaj, Peter Matthew Belford, Benjamin N. Morris, David X. Zhao, Saraschandra Vallabhajosyula

**Affiliations:** 1 Department of Medicine, Barnes-Jewish Hospital, Washington University of Saint Louis, St. Louis, MO 63110, USA; s.venkataraman@wustl.edu; 2 Respiratory Institute, Cleveland Clinic Foundation, Cleveland, OH 44106, USA; bhardwa3@ccf.org; 3 Section of Cardiovascular Medicine, Department of Medicine, Wake Forest University School of Medicine, Winston-Salem, NC 27157, USA; belford@wakehealth.edu (P.M.B.); dazhao@wakehealth.edu (D.X.Z.); 4 Section of Cardiovascular and Critical Care Anesthesia, Department of Anesthesiology, Wake Forest University School of Medicine, Winston-Salem, NC 27157, USA; bmorris@wakehealth.edu

**Keywords:** extracorporeal membrane oxygenation, myocarditis, mechanical circulatory support, cardiogenic shock, cardiac intensive care unit

## Abstract

Fulminant myocarditis is characterized by life threatening heart failure presenting as cardiogenic shock requiring inotropic or mechanical circulatory support to maintain tissue perfusion. There are limited data on the role of veno-arterial extracorporeal membrane oxygenation (VA-ECMO) in the management of fulminant myocarditis. This review seeks to evaluate the management of fulminant myocarditis with a special emphasis on the role and outcomes with VA-ECMO use.

## 1. Introduction

Myocarditis is defined as inflammation of the myocardium, generally following an injury due to but not limited to ischemia, infection, or trauma, diagnosed by established histological, immunological, and immune-histochemical criteria [1]. Fulminant myocarditis is a specific clinico-pathological form of myocarditis characterized by life threatening heart failure presenting as cardiogenic shock requiring inotropic or mechanical circulatory support (MCS) to maintain tissue perfusion [2,3]. Animal models with mice show that the disease process usually involves an initial active inflammatory state which can be caused by direct cytotoxicity by pathogens or cytokines released due to a similar pathogen [4,5]. In these models, mice with fulminant myocarditis seemed to have a marked elevation of cytokines with negative inotropy-like interferon-γ and tumor necrosis factor-α. These cytokines then triggered T-effector cells to cause persistent myocardial depression which is reversible. Temporary mechanical circulatory support (MCS) systems help in bridging over this period of myocardial depression prior to myocardial recovery [4].

Findings of acute myocarditis can mimic those of ischemic heart disease, where both conditions can have increasing natriuretic peptides [1] and diffuse ST-T-segment changes and regional wall motion abnormalities [6]. The use of endomyocardial biopsy (EMB) has helped with histopathological diagnosis where features of inflammation secondary to direct cytotoxic effects can be seen.

## 2. Epidemiology

Although fulminant myocarditis is relatively uncommon [7,8] and only known to be present in 10–15% of patients with myocarditis, the recent increase [9] in the cases of myocarditis and the severity associated with the illness make it relevant. The global burden of disease study [10] estimates the incidence of myocarditis to be around 2.48 million with a 18.7% increase between 2006 and 2016, suggesting that the disease is becoming more common even without considering the effects of the latest coronavirus disease 2019 (COVID-19) pandemic. As expected, patients with fulminant myocarditis are at an increased short term mortality risk driven by a more severely impaired left ventricular ejection fraction (LVEF) and arrhythmia burden [11]. Ammirati et al. [12] studied 443 patients with acute myocarditis and observed that patients (26.6%) with reduced (<50%) LVEF, ventricular arrhythmias, or a low cardiac output syndrome had higher overall short-term mortality when these patients were compared to those without a reduced LVEF and other complications. In contrast, when examining long-term trends of acute myocarditis (viral or autoimmune) compared to fulminant myocarditis, multicenter studies [5,12,13] show a better prognosis for fulminant myocarditis as they are more likely to experience complete recovery. McCarthy et al. [13] compared fulminant myocarditis with acute myocarditis (absence of vasopressor/inotrope use or left ventricular assistance) where they identified 147 patients considered to have myocarditis based on EMB and the Dallas histopathological criteria [14]. During a long-term median follow-up of 5.6 years of the 15 patients with fulminant myocarditis, 93% did not need heart transplantation compared to 45% of those with acute myocarditis. The difference in prognostic risk likely stems from the etiology of the illness, where fulminant myocarditis usually has an acute etiology with high short-term mortality and improvement following resolution of the acute stressor when compared to non-fulminant myocarditis which can have long-term etiologies. 

## 3. Diagnostics

Given the overlap of signs and symptoms with multiple other cardiac conditions, myocarditis is a diagnosis made with a combination of imaging, biomarkers, and EMB.

### 3.1. Biomarkers

Although inflammatory markers such as C-reactive protein and the erythrocyte sedimentation rate are elevated in myocarditis, they are mostly non-specific. Even cardiac specific markers such as troponin and brain natriuretic peptides are relatively non-specific and do not help in the definitive diagnosis of myocarditis [6]. Cardiac specific antibodies and autoantigens can be useful in the diagnosis of myocarditis in the absence of immunomodulation. Examples include anti-beta1-AR, anti-beta2-AR, anti-muscarinic acetylcholine receptor-2 among many others [15]. Although viral antibodies can be helpful in etiological diagnosis, they are not useful for the diagnosis of myocarditis. 

### 3.2. Imaging

Echocardiography is useful in diagnosing global ventricular dysfunction, diastolic dysfunction, and regional wall motion abnormalities that are often seen myocarditis in addition to a non-dilated, thickened, and hypo-contractile left ventricle seen in fulminant myocarditis. 

Cardiac magnetic resonance (CMR) imaging remains the most important modality for diagnosis [16]. The Lake-Louise criteria [17] are used for diagnosis where if at least 2 of the following are present confirm diagnosis: 

There is an increased global myocardial early gadolinium enhancement ratio between myocardium and skeletal muscle in gadolinium-enhanced T1-weighted images.

There is a regional or global myocardial signal intensity increase in T2-weighted oedema images.

There is at least one focal lesion with non-ischemic regional distribution in inversion recovery-prepared gadolinium-enhanced T1-weighted images (late gadolinium enhancement).

#### 3.2.1. Endomyocardial Biopsy (EMB)

The EMB has slowly gained traction in being the gold standard in the management of myocarditis [5,18]. A biopsy is indicated in patients with unexplained myocarditis with the following characteristics [6,19]: Mobitz type 2 s degree or higher heart block, persistent and refractory ventricular tachycardia, and heart failure requiring inotropic or MCS. 

Following histopathological examination, myocarditis can be further subclassified as [20]: 

Inflammation Positive/Virus Positive

Inflammation Positive/Virus Negative

Inflammation Negative/Virus Positive

Inflammation Negative/Virus Negative

#### 3.2.2. Deleterious Effects of Circulatory Shock in Myocarditis

The combination of high arrhythmia burden, rapid bi-ventricular failure, and concurrent multiorgan failure makes fulminant myocarditis the most severe form of myocarditis and is associated with a much higher mortality [6]. Overall rates of mortality and heart transplantation in fulminant myocarditis patients were found to be 28% among 165 fulminant myocarditis patients [21]. This is lower compared to the nearly 50% in-hospital mortality [22] seen with cardiogenic shock with myocardial infarction, but mortality rates among those with fulminant myocarditis are only estimates due to a lack of definitive studies. 

## 4. Management

**Figure 1:**
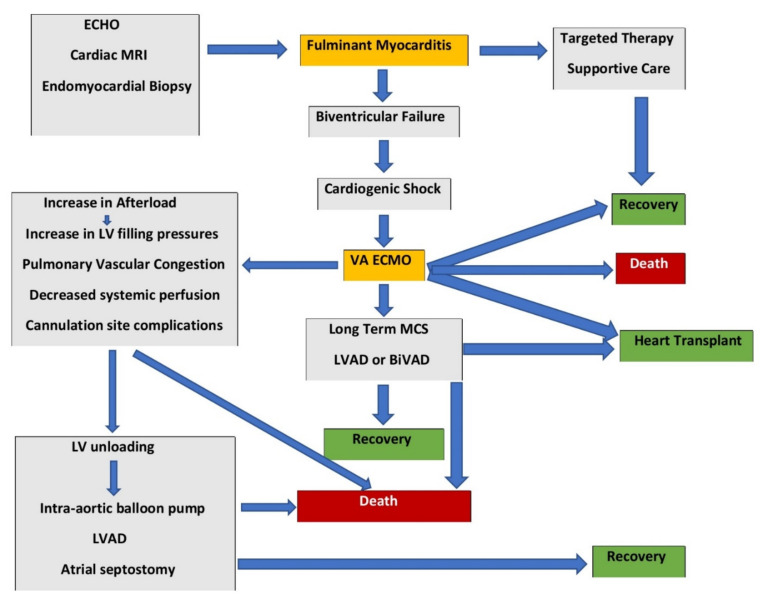
Fulminant myocarditis management and the role of veno-arterial extracorporeal membrane oxygenation.

The management of fulminant myocarditis as outlined by the European [6] and American societies [19] is mostly based on expert opinion due to a lack of reliable evidence in the management of fulminant myocarditis (Figure 1). 

### 4.1. Etiology Targeted Therapy

Causes of myocarditis can be classified as infectious and non-infectious (Table 1). In patients who are inflammation positive, they can be further sub-classified as: eosinophilic, lymphocytic, giant cell, and sarcoid. Immunosuppressive agents are known to be helpful in treating virus negative, inflammation positive chronic myocarditis. Escher et al. [23] studied 114 patients diagnosed with virus negative chronic myocarditis on EMB and treated with prednisone and azathioprine for 6 months and were followed for 10 years. Patients on immunosuppression were seen to have a significant improvement in LVEF compared to baseline after the 6-month period where it increased from 44.6 ± 17.3 to 51.8 ± 15.5%. Long-term follow-up also showed a marked improvement. A similar study was conducted by Cooper et al. [24] where patients with giant cell myocarditis were studied prospectively for the effects of immunosuppression, and they observed that steroids and cyclosporine markedly improved long-term survival.

#### 4.1.1. Initial Support

Initial stabilization can require both circulatory and respiratory support, and in the setting of fulminant myocarditis, likely including vasoactive medications. Although patients on norepinephrine were found to have fewer arrhythmias than those on dopamine, there are no studies to compare these effects in those with myocarditis [25]. Similarly, although milrinone and dobutamine were found to have similar composite outcomes with regard to in-hospital death, nonfatal myocardial infarction, transient ischemic attack, stroke, resuscitated cardiac arrest, receipt of a cardiac transplant, or MCS [26], it remains to be seen if this study can be directly extrapolated to a population with fulminant myocarditis. 

Both brady- and tachyarrhythmias are very common in acute myocarditis as an inflammatory milieu increases the occurrence of re-entrant ectopic foci [27,28]. Ventricular arrhythmias are known to be commonly associated with myocarditis [28], although patients can also develop supraventricular tachycardias. Amiodarone can be used to treat myocarditis induced arrhythmias [29]. Due to a high likelihood of recovery following the reversal of the acute phase, implantable cardiac defibrillator (ICD) placement should be deferred until the determination of LV function status. Although rare, some patients have a high arrhythmia burden with normal LV function; these patients should be evaluated for ICD placement on a case-by-case basis [6].

Role of mechanical circulatory support.

Patients with refractory shock despite pharmacological support require escalation to MCS. Currently, there is no clear consensus on optimal MCS for the care of patients with myocarditis. In patients without biventricular failure, patients can be adequately treated with initial temporary percutaneous uni or biventricular transvalvular axial pump based circulatory support. In a study by Annamalai et al. [30], 34 patients received Impella 2.5, CP, or the RP device in fulminant myocarditis. Of the 34 patients, 15 completely recovered while 1 patient required a heart transplant and 10 required additional MCS. The authors concluded that the Impella was safe and provided effective hemodynamic support to promote myocardial recovery. 

Veno-arterial extracorporeal membrane oxygenation (VA-ECMO).

Ventricular assist devices or veno-arterial extracorporeal membrane oxygenation (VA-ECMO) are predominantly being used in contemporary practice but are associated with higher mortality [31]. Although VA-ECMO is known to be associated with higher mortality risk in comparison with other MCS devices [32], registry based studies show that biventricular failure patients are most frequently initially treated with extra-corporeal membrane oxygenation (ECMO) [33,34]. Several institutions have successfully used mechanical circulatory support devices including VA-ECMO in the management of fulminant myocarditis [35,36,37,38]. Significant technological advances allowed VA-ECMO to be more portable with broader application. In the early 1970s, it was predominantly used in the resuscitation of neonates with respiratory failure. By the 1980s, VA-ECMO was used in pediatric cardiac arrest, and by the 1990s, ECMO use was extended to adult respiratory and cardiac failure. Contemporary data from the United States show a significant uptake in the use of VA-ECMO in cardiogenic shock from multiple etiologies—acute myocardial infarction, post-cardiotomy, biventricular tachycardia storm, and other etiologies [39,40,41,42,43,44]. Furthermore, since patients with fulminant myocarditis can rapidly decompensate from cardiogenic shock into cardiac arrest, VA-ECMO can be used in the resuscitation of the patients during cardiac arrest and subsequently can act as a bridge to recovery or transplant [45].

MCS devices may serve as a bridge to recovery, durable left ventricular assist devices (LVAD), or cardiac transplantation. Following initial stabilization during hyperacute decreases in cardiac power, MCS devices assist by allowing time for anti-inflammatory strategies to act [46]. These patients typically have biventricular failure, necessitating either biventricular MCS or VA-ECMO. While biventricular temporary VADs offer the option of direct unloading without an increase in filling pressures, they do not provide oxygenation (respiratory) support and are often prohibitive from a cost standpoint [47]. On the contrary, VA-ECMO is associated with higher LV afterload, thus worsening wall stress, increased oxygen demand, and an increase in inflammatory responses, though LV venting can mitigate the problem [20]. However, the VA-ECMO has remained popular in smaller centers due to cost of use and availability as compared to other VADs but still requires specialized personnel for bedside management [34].

Over the last two decades, there has been a tremendous growth in the number of centers offering ECMO in the US. According to ELSO, there are 521 medical centers in the US offering ECMO as of 2020 [48]. It is important to recognize that despite this growth and use of ECMO in developed nations, the cost of this therapy limits its use in low- and middle-income countries. For example, Mishra et al. [49] reported the cost analysis of ECMO use from Norway and highlighted that the mean cost of the ECMO procedure was USD 73,122 (SD of 34,786), and the mean total cost of the hospital stay was USD 213,246 (SD of 12,265). Chung et al. [50] provided the age-based cost of ECMO use and noted that a younger cohort (age 18–49), due to a longer hospital length of stay, had the highest median hospitalization cost at USD 147,548 (IQR 77,943–263,958) and was lowest in the age group of 80–90 years at USD 105,350 (IQR 71,147–151,906). More recently, Hayanga et al. [51] provided an in-depth analysis of the cost of ECMO based on an indication using the Nationwide Inpatient Sample. As expected, patients waiting for a heart transplant have a longer hospital length of stay and ECMO days and as a result have a higher associated cost of USD 1,448,931, while patients with cardiogenic shock who recover have a lower length of stay and an associated cost of USD 655,099. The authors conclude that over the years, the cost of overall charges for ECMO are increasing. With regard to the cost efficacy of ECMO in FM, we do not have any specific data at this stage, and future studies should consider reporting quality adjusted life years and the cost of ECMO associated hospitalization.

One of the major utilities of VA-ECMO therapy is in fulminant myocarditis refractory to pharmacological therapy, intra-aortic balloon pump support, and Impella therapy. Registry-based studies [33,46] have explored the use of ECMO in adults as the first line of therapy for fulminant myocarditis. In a combined adult and pediatric population, Hsu et al. [33] explored the utility of VA-ECMO in patients with profound, rapidly progressive ventricular dysfunction and needed maximal inotropic support. In this retrospective cohort of 75 patients, 48 patients (64%) survived on discharge with six requiring durable left VAD. Although it was a retrospective observational study, the authors concluded that mortality among fulminant myocarditis is lower, and VA-ECMO can be used as a first line of therapy in these patients. 

Saito et al. [52] charted outcomes of 30 fulminant myocarditis patients, 23 of which used ECMO as first-line MCS. Early conversion to temporary VAD prior to the elevation of total bilirubin had markedly improved mortality as compared to patients persistently on percutaneous ECMO. 

The pediatric population has also been studied extensively due to the high incidence of fulminant myocarditis. Rajagopal et al. [53] followed pediatric patients between the ages of 3 to 96 months with a diagnosis of severe cardiorespiratory failure due to myocarditis for 12 years. They observed a 61% survival similar to that of adults. Per the Extracorporeal Life support Organization (ELSO) registry report, of the 6225 pediatric patients needing VA-ECMO support for myocarditis, 65% were weaned successfully, but only 49% survived to hospital discharge [54].

Clinical outcomes with veno-arterial extracorporeal membrane oxygenation.

Ammirati et al. [11] observed that patients with fulminant myocarditis have a higher mortality and heart transplantation needs when compared to those with non-fulminant myocarditis. Older studies noted around 70% survival in patients with fulminant myocarditis needing VA-ECMO support [55,56]. Lorusso et al. [34] recently described a multicenter study analyzing fulminant myocarditis patients treated with VA-ECMO during a 5-year follow-up period. In their study population, 57 patients with the diagnosis of fulminant myocarditis were treated with VA-ECMO; 47 patients had a peripheral approach, and 10 patients had a central approach. Patients were supported with VA-ECMO for a mean duration of 9.9 ± 19 days, where 75.5% recovered with ECMO weaned successfully, and 71.9% survived to discharge. This was one of the first studies to follow this population longitudinally and found that 5-year survival among adults was 65.2 ± 7.9%, with recurrent self-recovering myocarditis observed in two patients. 

Chong et al. [57] studied 35 patients with fulminant myocarditis to determine demographics, hemodynamics, and labs of survivors when compared to non-survivors. They observed that pre-ECMO lactate and troponin-I levels were associated with a higher mortality in patients with fulminant myocarditis requiring VA-ECMO support. Similarly, Lee et al. [58] attempted to define the patient population that was more likely to survive by retrospectively studying 33 children. They also observed that the Pre-ECMO lactate level was associated with mortality. In this study, they also determined LVEF in all patients prior to ECMO and found that survivors had similar LVEF compared to non-survivors (38% vs. 33%). In contrast, post-ECMO was significantly different in the two groups with survivors having an EF of 56% as compared to non-survivors with an EF of 34.6% (*p*: 0.001). These studies are summarized in Table 2.

Most recently, Lee et al. [60] analyzed risk factors in pediatric patients diagnosed with fulminant myocarditis treated with VA-ECMO. They showed that among 71 patients who underwent ECMO, use of creatinine kinase-MB (CK-MB) with a cut-off of 94.74 ng/mL and a SOFA score with a cut-off score of 12 accurately predicted mortality. Given the ease of use of all the above markers, the use of lactate, cardiac enzymes (troponin-I, CK-MB), and LVEF remains viable.

#### 4.1.2. Management Approach 

For management of fulminant myocarditis, both peripheral and central approaches to VA-ECMO cannulation have been studied. When compared to peripheral VA-ECMO, there are clear advantages of central cannulation including offloading of the LV thereby preventing pulmonary edema and progression of the myocardial inflammation. However, these advantages come at the cost of higher complication rates compared to peripheral VA-ECMO. As a result, most centers prefer peripheral cannulation and if needed transition to central cannulation. Asaumi et al. [59] compared the outcomes of 14 patients with fulminant myocarditis on percutaneous VA-ECMO with those of 13 patients with non-fulminant myocarditis, demonstrating 70% survival in the fulminant myocarditis compared to no deaths in the non-fulminant myocarditis group cohort.

More recently, Tadokoro et al. [61] showed that conversion from peripheral to central extracorporeal life support (ECLS) is safe and feasible. Authors reported their experience of following 70 patients with fulminant myocarditis over 16 years who were managed by temporary MCS. Of the 70 patients in the study, 48 patients were transitioned from peripheral ECMO to central ECMO surgically. Authors concluded that although there was no significant difference in 5-year survival between peripheral vs. central ECMO, there was more pulmonary edema and multi organ failure in patients with central ECMO. Furthermore, only 62% of patients were weaned from central ECMO compared to 95% of peripheral ECMO [61]. A durable LVAD was implanted in patients who failed weaning from central ECMO as a bridge to transplant. Hence, although central cannulation is feasible, the percutaneous approach is more appropriate in the setting of acute decompensation.

#### 4.1.3. Timing of ECMO Initiation

For patients with fulminant myocarditis, the exact timing of ECMO initiation is not clear. There are no established guidelines or consensus on the exact timing of ECMO initiation, since timing is often driven by the hemodynamic status of the patient, institution specific policies, and local expert opinions/availability. Early cannulation and initiation of ECMO in an unstable patient are favored based on studies that provided data for time to cannulation. For example, Asaumi et al. reported that the median time between the onset of heart failure and ECMO initiation was 15 (12–20 h), range 7–36 h [59]. Similarly, Diddle et al. reported 61% survival to hospital discharge for patients with acute myocarditis, and the time to ECMO initiation was 13.5 h (3.5–24.5 h) [62]. Previously, several studies showed an ‘earlier the better’ approach in cardiogenic shock and ECMO timing [63,64,65,66]. Recently, Lee et al. reported the findings of the timing of ECMO in cardiogenic shock and outcomes. Authors divided the patient cohort based on time from shock to ECMO into three groups of early (<0.9 h), intermediate (1–2.2 h), and late (>2.2 h). Early ECMO (0.6 h) was shown to be associated with improved outcomes in patients with refractory cardiogenic shock when compared to intermediate (1.4 h) or late ECMO initiation (5.1 h). We recognize that even in the late group, the timing to ECMO initiation is within the first 6 h [67].

#### 4.1.4. Escalation of Care

The Lombardy registry which is a multicenter registry that included 443 patients with a diagnosis of acute myocarditis concluded that patients with LVEF <50% on the first echocardiogram, and/or sustained ventricular arrhythmias (VA), and/or hemodynamic instability on admission, had a higher mortality and were likely to require MCS [12]. Although observational studies and meta-analyses show no improvement in survival with the use of pulmonary artery catheters [68,69], recent single-center observational data show benefits in the setting of acute myocardial infarction and acute decompensated heart failure. It can be useful in patients with fulminant myocarditis to determine worsening filling pressures, vascular resistance, and cardiac indices in the ICU [70,71,72]. Furthermore, recent AHA guidelines recommend hemodynamic monitoring with right heart catheterization [73].

In addition to hemodynamic monitoring, serial assessment of end-organ perfusion is also necessary. Fuernau et al. [74] observed that arterial lactate measured after 8 h of MCS insertion with a cut-off value of 3.1 mmol/L showed the best discrimination for prognosticating patients in cardiogenic shock. Additionally, cardiac power output (CPO) and the pulmonary arterial pulsatility index (PAPi) with cut-offs were determined to be 0.6 W and >1.0, respectively [75].

#### 4.1.5. Left Ventricular Unloading

Although ECMO is most used, there exist multiple limitations with its use. These include an increase in afterload leading to elevated LV filling pressures, pulmonary vascular congestion, decreased systemic (renal and hepatic) perfusion, and cannulation site complications [76,77,78,79,80]. Most importantly, the increase in afterload caused by ECMO increases myocardial stress and increases the inflammatory milieu, thus decreasing the likelihood of recovery in conditions such as myocarditis [23]. To offset the increase in afterload and concurrent myocardial strain, LV unloading using intra-aortic balloon pumps, percutaneous LVAD, or atrial septostomy may be utilized. Additional right sided percutaneous VADs may be used to offload the right ventricle as needed [43,81,82].

#### 4.1.6. ECMO-IABP Strategy

An Intra-aortic balloon pump (IABP) can also be used to offload the left ventricle by partially offsetting the afterload increase caused by ECMO. Bakhtiary et al. [83] studied VA-ECMO as a treatment option in cardiogenic shock and saw that placement of an IABP independently reduced mortality among the 30 patients with dual support of ECMO and IABP. In the largest meta-analysis on this combination of MCS, our group evaluated the use of concomitant IABP in all-comer patients with CS receiving VA-ECMO [43]. This study did not note any additional survival benefit from VA-ECMO + IABP in the overall population but showed a survival advantage in the acute myocardial infarction population. Whether a similar benefit can be seen in the myocarditis population remains to be studied.

#### 4.1.7. ECMELLA (ECMO-Impella) Strategy

This strategy is used in cases with biventricular failure seen commonly in fulminant myocarditis where the afterload increase caused by VA-ECMO is offset by a left sided Impella. Impella devices have the added advantage of providing more hemodynamic support as compared to IABP devices while continuing to have the advantage of being percutaneous in the case of Impella 2.5 and CP. Pappalardo et al. [84] studied retrospective data from two tertiary critical care referral centers where VA-ECMO patients were compared to patients of ECMELLA. They found that patients on ECMELLA had a significantly lower mortality (47% vs. 80%) and a higher rate of successful bridging to recovery. Impellas are also successfully used in cases of fulminant myocarditis with more right sided failure than left in a case of giant cell myocarditis to bridge to a durable LVAD. The use of ECMELLA was previously assessed by our group [77] using a systematic review study design where we observed higher weaning from VA-ECMO and higher bridging to LVADs and heart transplant. Pappalardo et al. then described attempting to use two Impella devices instead of an ECMELLA with the aim of eliminating the afterload increase seen with ECMO [84]. They reported using Impella CP on the left and Impella RP on the right and called it the BIPELLA strategy.

#### 4.1.8. Veno-Arterial Extracorporeal Membrane Oxygenation Weaning

Given the global deficit of available heart donors [85], a process to determining factors that predict weaning from ECMO becomes more important. Matsumoto et al. [86] attempted to determine factors predictive of successful weaning by investigating 37 fulminant myocarditis patients on ECMO. During a follow-up period of 48 months, when 22 patients who were successfully weaned were compared to the 15 who could not be weaned successfully, the authors found significant differences between the two groups’ levels of creatinine kinase, LV posterior wall thickness, and the presence of arrhythmias on admission and the first three days on ECMO. Of the 15 who could not be weaned from ECMO, few survived despite the transition to VAD. They concluded that a clinical profile that characterizes dysrhythmia burden, cardiac injury with biomarkers, and echocardiographic changes can accurately identify patients who cannot be weaned from ECMO successfully.

Jaroszewski et al. [87] implemented a triple stage bridge where fulminant myocarditis patients on ECMO were initially transitioned to a short-term bi-VAD; this was followed by a transition to a long-term para-corporeal VAD that was weaned completely after total recovery.

To prevent thrombosis in the ECMO circuit, several guidelines recommend the use of continuous systemic anticoagulation [88]. However, despite systemic anticoagulation, the incidence of circuit thrombosis was reported in 15.6% of the patients on VA ECMO with a venous thromboembolism incidence of 10% [89,90]. In a recent systemic review by Olson et al. [91], the authors found that the incidence of thrombosis in anticoagulation free ECMO was comparable to those with systemically anticoagulated ECMO [78,80]. The authors found circuit thrombosis in 13.4% of the patients on VA ECMO with a venous thromboembolism incidence of 9.5% [89]. Not surprisingly, bleeding is the most frequent complication of ECMO affecting as many as 30–50% of the patients [92,93,94]. Further, Aubron et al. highlighted that bleeding is independently associated with a higher incidence of in-hospital mortality [95]. Recognizing that the burden of bleeding and thrombosis is very high in ECMO patients and is associated with poor outcomes, future studies should consider analyzing data accounting and adjusting for these complications when reporting outcomes of patients with FM on ECMO.

Long-term consequences of mechanical circulatory support in fulminant myocarditis.

Mirabel et al. [55] analyzed fulminant myocarditis patients that survived hospitalization requiring VA-ECMO support, with short form scores (that measure overall health status) to evaluate quality of life measures, and showed that survivors had satisfactory mental health and vitality but persistent physical and psychosocial-related defects. They also found that 38% had anxiety and 27% had persistent depression when compared to age- and sex-matched controls. This is similar to the incidence seen among patients with refractory cardiogenic shock who survived hospitalization requiring VA-ECMO [96].

Den Uil et al. [97] conducted a systematic review of MCS device use between 2006 and 2016 and found that patients on ECMO had a median support time of 6–7 days. In patients with myocarditis, if there were no signs of recovery by 14 days, they were likely bridged to LVAD or heart transplant. Patients who continued to have low LVEF (<39%) at 14 days had a high risk of mortality and should be considered for LVAD therapy or heart transplants [58].

In cases without myocardial recovery, ECMO stabilizes and optimizes cardiogenic shock prior to semi-urgent cardiac transplantation [98]. Multiple case-reports of fulminant myocarditis [99,100] describe successful acute heart transplants in patients who could not be weaned off ECMO. Observational retrospective studies showed that although patients who receive transplant have a relatively high mortality rate, if they do receive a transplant, they have a very high survival rate. Hsu et al. [33] showed that among the three patients who underwent heart transplant, all survived. Ting et al. [101] studied 134 patients with fulminant myocarditis requiring MCS; of these, six patients on ECMO underwent transplant, and four survived. In the population they studied, sepsis was the most common cause of death following transplant.

## 5. COVID-19, Myocarditis, and Extracorporeal Membrane Oxygenation

Since the start of the COVID-19 pandemic in March 2020, acute myocarditis caused by SARS-CoV-2 has been recognized as one of the leading cardiovascular complications of COVID-19 disease. During early stages of the pandemic, in 16 patients, Cinar et al. [102] noted that acute myocarditis was predominantly seen in male patients with a history of hypertension, and the electrocardiographic findings were mostly nonspecific. Furthermore, the diagnosis of acute myocarditis was made without EMB in all the patients, and CMR imaging was used in only three of the cases for the diagnosis. Over the last year and a half, there has been a constant growth in the number of the case reports of acute myocarditis caused by SARS-CoV-2 in the literature [103,104]. More recently, there were some concerns with COVID-19 vaccine being associated with myocarditis and pericarditis [105,106]. For example, Diaz et al. recently reported the incidence of post COVID-19 vaccination associated with myocarditis to be at 1 in 100,000 [106]. Similar reports were published earlier by Montgomery et al. with myocarditis in 23 military personnel out of 2.8 million vaccines administered [105]. In light of the current COVID-19 pandemic, the role of ECMO as a bridge to recovery from respiratory failure and as a bridge to lung, heart, and or combined heart-lung transplant in the event of refractory respiratory failure and/or cardiogenic shock has been acknowledged by the Extracorporeal Life Support Organization (ELSO) [107] and American Society for Artificial Internal Organs (ASAIO) [108]. Since fulminant myocarditis caused by SARS-CoV-2 or otherwise is a potentially reversible cause of acute heart failure and cardiogenic shock, MCS using ECMO is recommended while the myocardial inflammation subsides.

Although, the definitive pathophysiology of the myocarditis caused by SARS-CoV-2 is not currently well established, several mechanisms are proposed based on the understanding of other viral diseases associated with myocarditis. For example, Bojkava et al. demonstrated that SARS-CoV-2 can cause direct cellular damage once it gains entry into the cardiac myocyte using the angiotensin converting enzyme receptors or indirectly by mounting a T-cell mediated cytotoxic immune response as shown by Gauchotte et al. [109,110]. An innate or acquired immune mediated response to the virus can lead to apoptosis and necrosis, and autoimmune injury in response to the virus induced injury has also been proposed [111]. Lastly, another proposed mechanism of myocarditis is a cytokine storm associated with the release of tumor necrosis factor alpha, and interleukins 2, 6, and 10 [112].

Although COVID-19 disease predominantly leads to respiratory failure, and these patients can be managed with veno-venous ECMO alone, patients who develop fulminant myocarditis have concomitant biventricular failure. VA-ECMO is therefore the circuit of choice due to refractory cardiogenic shock while allowing time for myocardial inflammation to subside. Zeng et al. reported the first case of fulminant myocarditis requiring ECMO support and highlighted the concern for cardiac complications of COVID-19 [113]. Papageorgious et al. reported the first case of biopsy proven fulminant myocarditis in a patient with COVID-19 requiring VA-ECMO [114]. Marcinkiewicz et al. and Yeleti et al. also highlighted the role of VA-ECMO in this patient population with both patients requiring temporary circulatory support with VA-ECMO and eventually full recovery [115,116].

### Prognosis

Patients with fulminant myocarditis and COVID-19 carry high mortality not only because of myocarditis in isolation but also because several of these patients have multi-organ failure including acute respiratory and renal failure. As previously discussed, several patients required ECMO support to manage fulminant myocarditis after COVID-19. Occasionally some patients may demonstrate recovery with supportive care [117]. On the other hand, where there are no clinical or echocardiographic signs of recovery, VA ECMO is used as a bridge to heart transplant [29]. Currently, there are limited long-term data on the management and outcomes of fulminant myocarditis in COVID-19.

## 6. Conclusions

In conclusion, fulminant myocarditis has a low incidence but continues to be associated with poor outcomes in the contemporary era. MCS devices, such as VA-ECMO, provide adequate circulatory support and may serve as a bridge to recovery or definitive therapy with heart transplant or durable VADs. Due to the limited incidence of this disease state, there remain multiple avenues for future research in the diagnosis, management, and prognosis of fulminant myocarditis.

## Figures and Tables

**Table 1:** Etiology of fulminant myocarditis.

Infectious	Non-Infectious
Bacterial Staphylococcus, Streptococcus, Meningococcus, Gonococcus, Salmonella, Hemophilus influenza, Mycoplasma pneumonia, Brucella	Toxin mediated
Spirochetal Borrelia, Leptospira	Physical Radiation induced; Electric shock induced
Viral DNA viruses: human herpes virus–6, Epstein-Barr virus, varicella-zoster virus, herpes simplex virus, adenoviruses, parvovirus B19, cytomegalovirus RNA viruses: coronaviruses, respiratory syncytial virus, mumps virus, measles virus, rubella virus, hepatitis C virus, dengue virus, yellow fever virus, Chikungunya virus, coxsackieviruses A and B, echoviruses, polioviruses, influenza A and B viruses	Drugs Cocaine, cyclophosphamide, ethanol, fluorouracil, lithium, catecholamines, hemetine, interleukin, amphetamines, anthracyclines, trastuzumab
Fungal Cryptococcus, Histoplasma, Aspergillus, Actinomyces, Blastomyces, Candida, Coccidioides	Heavy metals Lead, copper, iron, arsenic
Parasitic Echinococcus granulosus, Taenia solium, Trichinella spiralis	Hormonal Pheochromocytoma
Rickettsial Coxiella burnetii, R. rickettsii R. tsutsugamushi	Venoms Snake and spider bites, bee, and wasp stings
Protozoal Trypanosoma cruzi, Toxoplasma gondii, Entamoeba, Leishmania	Immune mediated
	Allergen Serum sickness, tetanus toxoid
	Antigen induced Infection-negative lymphocytic, infection-negative giant cell, heart transplant rejection

Abbreviations: DNA: Deoxyribonucleic acid, RNA: Ribonucleic acid.

**Table 2:** Survival rates fulminant myocarditis patients on VA-ECMO.

Author/Year	Study Design	Region	Total N	Configuration of VA-ECMO	Survival to Discharge	LVAD Transition	Long-Term Survival
Asaumi et al. 2005 [59]	Retrospective cohort	Japan	14	100% Peripheral	71%	---	71%
Hsu et al. 2011 [33]	Retrospective cohort	Taiwan	75	63% Peripheral 37% Central	64%	8%	---
Mirabel et al. 2011 [55]	Retrospective cross-sectional	France	35	80% Peripheral 4% Central 16% BiVAD	68%	---	---
Lorusso et al. 2016 [34]	Retrospective cohort	Italy	57	85.8% Peripheral 14.2% Central	75.5%	---	65.2%
Saito et al. 2018 [52]	Retrospective cohort	Japan	30	92% Peripheral 8% Central	83.3%	13%	---
Chong et al. 2018 [57]	Retrospective cohort	Taiwan	35	100% Peripheral	57%	---	55.6%

Abbreviations: BiVAD: biventricular assist devices; LVAD: left ventricular assist devices; MCS: mechanical circulatory support; VA-ECMO: veno-arterial extracorporeal membrane oxygenation.

## Abbreviations

CK-MB	creatinine kinase-MB
CMR	cardiac magnetic resonance
COVID-19	coronavirus disease 2019
ECLS	extracorporeal life support
ECMO	extracorporeal membrane oxygenation
ELSO	extracorporeal Life Support Organization
EMB	endomyocardial biopsy
ICD	implantable cardiac defibrillator
LVAD	left ventricular assist device
LVEF	left ventricular ejection fraction
MCS	mechanical circulatory support
SARS-CoV-2	severe acute respiratory syndrome coronavirus 2
VAD	ventricular assist devices
VA-ECMO	veno-arterial extracorporeal membrane oxygenation
